# Chemokine Receptor Profile of Circulating Leukocyte Subsets in Response to Acute High-Intensity Interval Training

**DOI:** 10.3390/biom16020263

**Published:** 2026-02-07

**Authors:** Katharina Leuchte, Sara Fresnillo Saló, Anne Rahbech, Mikkel Byrdal, Anders Vinther, Gitte Holmen Olofsson

**Affiliations:** 1 National Center for Cancer Immune Therapy (CCIT-DK), Department of Oncology, Copenhagen University Hospital Herlev, 2730 Herlev, Denmark; sara.fresnillo.salo@regionh.dk (S.F.S.); gitte.holmen.olofsson@regionh.dk (G.H.O.); 2 Department I of Internal Medicine, Medical Faculty and University Hospital of Cologne, University of Cologne, 50937 Cologne, Germany; 3 Department of Veterinary and Animal Sciences, Section of Biomedicine, University of Copenhagen, 1870 Frederiksberg, Denmark; 4 Department of Physiotherapy and Occupational Therapy, Copenhagen University Hospitals Herlev and Gentofte, 2730 Herlev, Denmark; 5 Department of Clinical Medicine, University of Copenhagen, 2200 Copenhagen, Denmark

**Keywords:** endurance exercise, acute exercise, exercise immunology, chemokine receptors, migration, homing

## Abstract

Physically active individuals demonstrate enhanced immune competence. Efficient execution of effector function relies on chemokine receptor-regulated immune cell trafficking along chemokine gradients to sites of inflammation, infection, tumors, or tissue damage. This study investigates the impact of acute high-intensity interval training (HIIT) on chemokine receptor expression in leukocytes. Sixteen healthy participants completed a single HIIT session, and peripheral blood was collected before exercise (Bsl), immediately after (Ex02), and one hour later (Ex60). Surface expression of selected chemokine receptors was measured using flow cytometry on CD4^+^ T cells, γδ T cells, NK cells, and monocytes, followed by FlowSOM clustering. NK cells, CD4^+^ T cells, and γδ T cells were strongly mobilized at Ex02 and returned to or below baseline at Ex60. HIIT preferentially mobilized CX3CR1^+^ CXCR2^+^ CD56^dim^ NK cells, CD4^+^ T cells expressing CX3CR1^hi^ and CCR5^+^, and CX3CR1^+^ CD56^+^ γδ T cells, indicating mobilization of immune cells phenotypically associated with migratory and cytotoxic potential. Proportions of intermediate and non-classical monocytes increased at Ex02 and decreased at Ex60. In conclusion, HIIT induced a rapid redistribution of leukocyte subsets with chemokine receptor profiles suggesting enhanced endothelial interaction and migratory capacity toward effector tissues.

## 1. Introduction

Strong evidence links physical activity to a longer life span and the prevention of various diseases [1]. The physiological response to high-intensity exercise relies on a complex interplay between different tissues—with the immune system playing an important role as a mediator [2,3]. One essential immunological response to exercise is the mobilization of leukocytes into peripheral blood [4]. Previous studies have highlighted how adrenergic signaling, through the release of the catecholamines epinephrine and norepinephrine, affects mobilization by binding to adrenergic receptors on immune cells [5,6]. Furthermore, this exercise-induced leukocytosis predominantly involves cells of higher differentiation and those with cytotoxic capacities [7]. However, less is known about how exercise impacts immune cell egression and, specifically, how it can be mediated by differential chemokine receptor expression.

Chemokine receptors, differentially expressed on the surface of various cells, are key regulators of cell trafficking along chemokine gradients. There are four chemokine receptor subfamilies, classified according to variations in the arrangement of two cysteine residues: CC, CXC, CX3C, and XC [8,9]. Chemokine receptors create a network with their ligands, reflecting a combination of specific and high receptor/ligand promiscuity. Under normal conditions, homeostatic chemokines maintain a dynamic tissue leukocyte composition and regulate cellular trafficking to prepare for immune responses. Examples of such are CCL21 and CCL19 recruiting CCR7^+^ T cells to the lymph node in search of cognate antigen; in the brain, CXCL12 mediates leukocyte homing via CXCR4 [10]. Chemokine receptors also influence immune cell recruitment to specific sites of inflammation, infection, tissue damage or tumors. Within the tumor microenvironment, chemokines can exert both pro- and anti-tumor effects by selectively recruiting immune cell populations according to their chemokine receptor expression. Examples are γδ T cells that exhibit high proportions of CCR5 expression (recruited by CCL5) [11]; monocytes also show expression of CCR5, together with CCR2; NK cells and CD8 are shown to express CXCR3 (receptor for CXCL9 and CXCL10), whereas CXCR2 is almost exclusively expressed on MDSCs together with CXCR4, which is also express by some dendritic cells (DCs) [8]. Other studies also suggest that CXCR6 has a key role in sustaining effector function of CD8^+^ cells in the tumor microenvironment [12]. Finally, CX3CR1 correlated with the degree of effector differentiation of T cells [13]. Beyond this, the activation of chemokine receptors can trigger a broad range of other biological processes, such as cell proliferation, survival, differentiation, cytokine secretion, degranulation, and the respiratory burst [8]. How exercise impacts chemokine receptor expression patterns has been sparsely studied, mostly in the context of obesity [14,15] and cancer [16,17].

We hypothesize that high-intensity interval training (HIIT), as a form of high-intensity endurance exercise, induces a shift in chemokine receptor expression, favoring immune cell subsets with enhanced migratory capacity to key sites of action, potentially improving immune surveillance and tissue repair with clinical relevance in contexts such as cancer, autoimmune and inflammatory disease, and infectious disease. While previous studies have examined exercise-induced lymphocyte mobilization, to our knowledge, this is the first study to comprehensively profile HIIT-induced changes in chemokine receptor expression across CD4^+^, γδ T cell, NK, and classical, intermediate and non-classical monocytes in healthy adults. Consequently, understanding the migratory potential of exercise-mobilized leukocytes, as indicated by their chemokine receptor expression profiles, is pivotal for elucidating their capacity for tissue infiltration and optimizing local immune surveillance.

## 2. Materials and Methods

### 2.1. Study Participants and Preparation Procedures

INHALE is an interventional study in healthy participants undergoing a high-intensity interval training (HIIT) session [18]. A detailed description of the INHALE study design can be found at clinicaltrials.gov (NCT05826496). Participants were free of exercise-limiting conditions and excluded if using medications affecting cardiovascular, metabolic, or immune responses (e.g., >10 mg prednisone equivalents or beta blockers). Health status and medication were evaluated by questionnaires. Written informed consent was obtained. Study design is shown in Figure S1. The study has ethics approval from the Capital Region’s Ethics Board (H-23006672) and the Danish Data Protection Agency (P-2023-95). Sixteen participants from the INHALE cohort (in total, 23) were selected for this study based on sample availability.

### 2.2. Cardiopulmonary Exercise Test

Participants completed a cardiopulmonary exercise test (CPET) on a cycling ergometer (CPET Vyntus CPX, Ergometer ViaSprint 150p, Vyarie Medical GmbH, Höchberg, Germany) following 24 h of avoiding alcohol, non-prescription meds, vigorous/unaccustomed exercise, and 2 h of caffeine fasting. Experienced physiotherapists estimated aerobic capacity based on participant information regarding habitual physical activity and exercise and selected appropriate incremental workload protocols aiming for a test duration of 8–12 min and terminated at volitional exhaustion [19]. Ventilation, heart rate (HR), VO_2_, and VCO_2_ were recorded every 15 s; respiratory exchange rate (RER), VO_2_ peak, maximal power output, and HR were calculated and confirmed visually by three independent researchers. For VO_2_ peak calculation, the average of two consecutive 15 s epochs with the highest VO_2_ was used. Anthropometric and body composition measurements by bioimpedance were performed at the CPET visit, separately from the exercise test.

### 2.3. Exercise Session and Blood Sampling

Within 24 days post-CPET, participants completed a physiotherapist-supervised, monitored, group-based exercise session after similar abstinence and fasting conditions, which took place during daylight between 8:00 and 14:00 to minimize bias by circadian rhythm. The warm-up included 5 min on a bicycle ergometer (Motion cycle 200 med, Emotion Fitness GmbH & CO, Hochspeyer, Germany) followed by 5 × 2 min circuit training on: (1) an air bike ergometer (ReNegaDE AIR BIKE C2, Evocardio, Hasselt, Belgium), (2) a ski ergometer (CONCEPT 2 Deutschland GmbH, Hamburg, Germany), (3) a rowing ergometer (Hiit Console, Core Health & Fitness LLC, München, Germany), (4) a cross trainer (motion cross 600, Emotion Fitness), and (5) exercises on a step bench. All warm-up exercises were self-paced but overseen by an experienced physiotherapist, encouraging the participants to exercise with sufficient intensity to substantially increase HR and respiration. Bicycle training started with 2 min of low-workload pedaling (approximately 50% of the maximal power output at CPET), followed by 3 × 3 min of high-intensity intervals alternating between 20 s of “all-out” (aiming at 100% of the maximal power output at CPET) and 20 s of easy pedaling (aiming at 50%). These intervals were separated by two 3 min steady-state sequences at 50–70% of the maximal power output during CPET. Power output and HR were manually registered during the session. Resistance was pre-set to meet or exceed target workloads. Only water was consumed until 60 min after the session.

### 2.4. Blood Sample Collection and Processing

Blood samples were collected at baseline (Bsl), within 2 min post-exercise (Ex02), and after 60 min (Ex60). Blood was drawn into Vacutainer Sodium Heparin Tubes (BD) and immediately transported by a dedicated staff member from the exercise facility to the sterile cell laboratory. Peripheral blood mononuclear cells (PBMCs) were isolated using density-gradient centrifugation (Lymphoprep, Stemcell Technologies, Vancouver, BC, Canada) in Leukosep tubes (Greiner Bio-One, Kremsmünster, Austria), counted, and stored in Cool Cell freezing containers (Corning Incorporated, Corning, NY, USA) at −80 °C within 2.5 h of venipuncture. The following day samples were transferred to long-term storage at −150 °C. All sample handling steps were performed by trained laboratory personnel according to a standardized protocol.

### 2.5. TBNK Analysis

Whole blood was stained with a 6-color TBNK panel (BD Biosciences, San Jose, CA, USA) in Trucount tubes according to the manufacturer’s protocol and acquired on a BD FACSCanto II. Gating was standardized (FACSCanto v.3.1.5878.21241 Software, BD).

### 2.6. Chemokine Receptor Staining

Cells were thawed in pre-warmed RMPI medium (Gibco, Grand Island, NY, USA), washed twice, and counted. One million cells were washed twice with PBS/2% FCS (FACS buffer) and stained in two sequential steps to account for different expression levels of the markers and improve detection sensitivity. First, cells were incubated with a chemokine receptor antibody mix at 37 °C for 15 min, followed by incubation with a lineage and differentiation marker antibody mix (Table S1) at 4 °C for 30 min. Cells were then washed twice, resuspended in FACS buffer, and acquired on the NovoCyte Quanteon (Agilent, Santa Clara, CA, USA). Dead cells were excluded by staining with Live/Dead Near IR Fixable Stain. Data was analyzed using FlowJo v10 and Novoexpress v1.5.0. The gating strategy is shown in Figure S2.

### 2.7. Flow Cytometry Data Analysis

Uniform Manifold Approximation and Projection (UMAP) analysis was performed on manually gated CD4^+^ T cells (CD3^+^, CD4^+^ CD8^−^), γδ T cells (CD3^+^, Vδ2^+^), NK cells (CD3^−^, CD56^+^), or monocytes (CD3^−^ CD14^+^), using 15 neighbors and a minimum distance of 0.5. The following markers were included for UMAP generation: CD28 and CCR7 in CD4^+^ T cells; CD56 in NK and γδ T cells; CD16 and CD14 in monocytes; and CX3CR1, CXCR4, CXCR2, CXCR6, CCR2, and CCR5 across all cell types. Unsupervised clustering was then conducted with the FlowSOM algorithm (grid size: 10 × 10) using the same markers. Clusters with less than 0.5% frequency were excluded from the analysis.

### 2.8. Statistics

This was an exploratory study; therefore, no power calculations were made. Given the exploratory design and sample size, the results are presented descriptively, without formal hypothesis testing. Data were analyzed and visualized using GraphPad Prism version 10 or R version 4.3.0 and RStudio 2024.04.2+764. Figures were created using Inkscape 1.3.2. Graphic figures were created using Biorender (https://www.biorender.com/). Before applying parametric or nonparametric methods, we checked for normal distribution using visual inspection of histograms or the Shapiro–Wilk test. Continuous variables are described as means and SD unless otherwise stated. Details are given in figure captions.

## 3. Results

### 3.1. Participant Characteristics and Exercise Program

We set out to assess chemokine receptor expression, as a response to one bout of supervised high-intensity exercise training (HIIT), on CD4^+^ T cells, γδ T cells, natural killer (NK) cells, and classical, intermediate and non-classical monocytes. Analyzed blood samples were collected before (Bsl), immediately after (Ex02), and one hour after (Ex60) exercise. A total of 16 participants from the INHALE study were included in this analysis; see study overview in Figure S1. Participants (11 males and 5 females) had a mean age of 31.7 years (25–65) and a mean BMI of 23 (20.3–26.8). The aerobic capacity of the participants was measured by their VO_2_ peak during the cardiopulmonary exercise test (CPET). Participants showed a VO_2_ peak of 39.7 mL/min/kg (29–47.3), reflecting a generally healthy population with varying levels of fitness. High intensity of the exercise intervention was verified by heart rate (HR) and power output (see Table 1). Overall, the INHALE study was designed to minimize confounding factors such as circadian rhythm, differences in blood sampling and processing, and variations in FITT criteria, and we applied a highly standardized, supervised HIIT protocol [18,20].

### 3.2. HIIT-Induced Changes in Chemokine Receptor Expression in NK Cells

**Figure 1:**
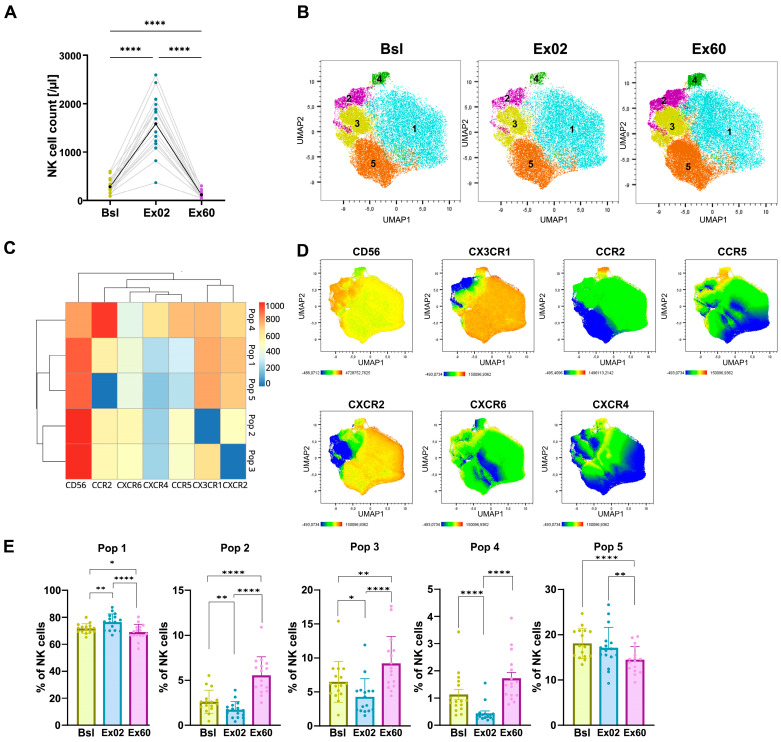
HIIT-induced mobilization of CD56^dim^ CX3CR1^+^CXCR2^+^ NK cells. (**A**) NK cell counts per µL of whole blood before (Bsl), immediately after (Ex02) and one hour after exercise (Ex60), using flow cytometric TBNK kit whole blood staining. (**B**–**E**) UMAP dimensionality reduction and FlowSOM unsupervised clustering were performed using a single integrated dataset comprising CD3^−^CD56^+^ cells from all participants and timepoints based on expression of CD56 and the chemokine receptors CCR5, CXCR2, CXCR6, CX3CR1, CCR2, and CXCR4. (**B**) UMAP visualization of the clusters identified via the FlowSOM clustering algorithm shown separately at Bsl, Ex02 and Ex60. (**C**) Heatmap showing the surface expression profiles across the five identified clusters. (**D**) UMAP overlaid with marker mean fluorescence intensity (MFI) of cells from all participants and timepoints. (**E**) Population frequency at Bsl, Ex02 and Ex60. Shown are individual values, and bar graphs show mean and SD. Percentage data were logit-transformed before being analyzed using repeated-measures ANOVA. Significance levels are indicated by asterisks on the graphs: * *p* ≤ 0.05, ** *p* ≤ 0.01, *** *p* ≤ 0.001, **** *p* ≤ 0.0001. N = 16.

We aimed to investigate leukocyte mobilization levels in our cohort following one bout of HIIT. To quantify absolute leukocyte numbers, a clinical-grade, standardized six-color TBNK kit was used at Bsl, Ex02 and Ex60. Acute HIIT profoundly mobilized NK cells (CD3^−^, CD56^+^, CD16^+^), resulting in a 6.6-fold NK cell increase in total counts (3.2–18.3, *p* < 0.0001; Figure 1A). One-hour post-exercise (Ex60), NK cell counts decreased 2.7-fold below baseline levels (0.8–6.9, *p* < 0.0001; Figure 1A).

To gain deeper insight into how exercise affects leukocyte migratory potential, we assessed surface chemokine receptor expression on the cells of interest. We analyzed both mean fluorescence intensity (MFI) and the percentage of receptor-positive NK cells for CX3CR1, CCR2, CXCR2, CCR5, CXCR4, and CXCR6 at all timepoints. Using FlowSOM unsupervised clustering, we first aimed to identify NK cell subpopulations based on chemokine receptor expression and then to compare these subpopulations across the three timepoints to determine whether all NK cells were mobilized to the same extent or whether a specific subpopulation was particularly sensitive to exercise-induced mobilization. Clustering revealed five distinct clusters defined by CD56 and chemokine receptor expression patterns (Figure 1B). We identified three clusters (Pop 1, Pop 4, and Pop 5) with CD56^dim^ expression, corresponding to a phenotype associated with higher cytotoxic potential [21]. On the other hand, Pop 2 and Pop 3 consisted of CD56^bright^, consistent with cytokine-producing NK cells. Acute HIIT preferentially mobilized Pop 1 from 71.7% of total NK cells at Bsl to 76.4% at Ex02. Pop 1 was characterized by CD56^dim^, high CX3CR1 and CXCR2 expression, and low CCR2 expression (Figure 1C–E). At Ex60, Pop 1 decreased below baseline values to represent 69.1% of total NK cells. Pop 5, CD56^dim^ with a similar chemokine receptor expression profile but lacking CCR2, decreased from 17.1% at Bsl to 14.49% at Ex60, consistent with egression to peripheral tissues. In contrast, Pop 2 and Pop 3, CD56^bright^ with low or absent CX3CR1 and CXCR2 expression, increased from 2.6% and 6.5% at Bsl to 5.5% and 9.2%, respectively, at Ex60, suggesting retention in circulation. Across all NK cell populations, CXCR4 and CXCR6 expressions were low. To assess potential sex-specific effects, we performed sex-stratified analyses. The observed dynamics showed no difference between females and males (Figure S3).

By analyzing single markers, the frequency of CX3CR1^+^ NK cells, CXCR2^+^ NK cells, and CXCR4^+^ NK cells was found to have increased at Ex02, suggesting a preferential mobilization (Figure S3).

### 3.3. HIIT-Induced Changes in Chemokine Receptor Expression in CD4^+^ T Cells

**Figure 2:**
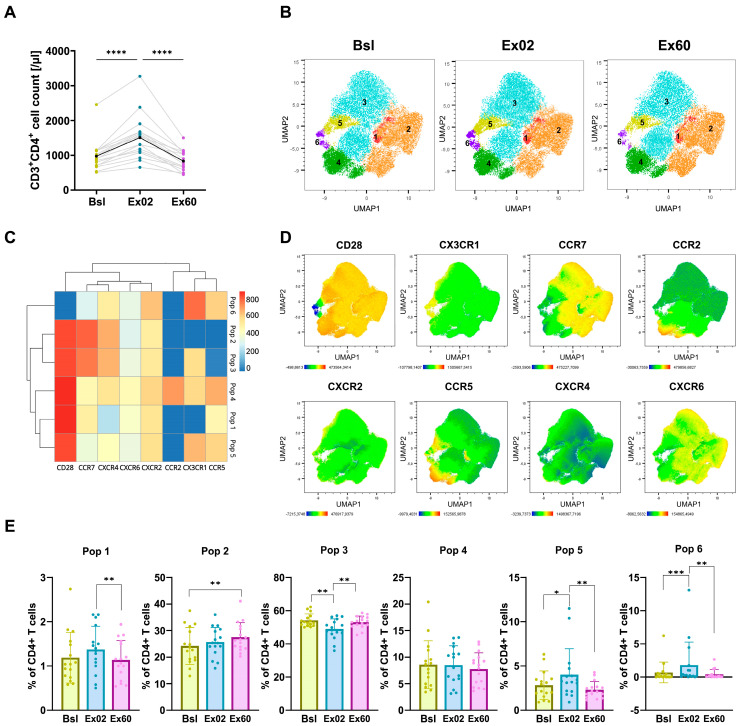
HIIT mobilizes CX3CR1^+^ CCR5^+^ CD4^+^ T cells. (**A**) CD4^+^ T cell counts per µL of whole blood before (Bsl), immediately after (Ex02) and one hour after exercise (Ex60), using flow cytometric TBNK kit whole blood staining. (**B**–**E**) UMAP dimensionality reduction and FlowSOM unsupervised clustering were performed using a single integrated dataset comprising CD3^+^CD4^+^ cells from all participants and timepoints based on expression of CD28 and the chemokine receptors CCR7, CCR5, CXCR2, CXCR6, CX3CR1, CCR2, and CXCR4. (**B**) UMAP visualization of the clusters identified via the FlowSOM clustering algorithm shown separately at Bsl, Ex02 and Ex60. (**C**) Heatmap showing the surface expression profiles across the six identified clusters. (**D**) UMAP overlaid with marker mean fluorescence intensity (MFI) of cells from all participants and timepoints. (**E**) Population frequency at Bsl, Ex02 and Ex60. Shown are individual values, and bar graphs show means and SD. Percentage data were logit-transformed before being analyzed using repeated-measures ANOVA. Significance levels are indicated by asterisks on the graphs: * *p* ≤ 0.05, ** *p* ≤ 0.01, *** *p* ≤ 0.001, **** *p* ≤ 0.0001. N = 16.

As key regulators of immune responses, exercise-induced changes in CD4^+^ T cells are of high interest. We therefore set out to investigate how chemokine receptors mediate CD4^+^ T cell recruitment to peripheral blood. In our study, CD4^+^ T cells were mobilized after acute HIIT with an increase of 1.5-fold (1.3–2.1, *p* < 0.0001) and subsequently decreased during Ex60 by 1.8-fold (1.3–2.8, *p* < 0.0001), returning to baseline levels (Figure 2A). Unsupervised clustering analysis revealed six distinct clusters based on expression of CD28, CX3CR1, CCR7, CCR2, CXCR2, CCR5, CXCR4 and CXCR6 (Figure 2B–D). Acute HIIT mobilized Pop 5 and Pop 6 preferentially at Ex02, which increased from 2.8% to 4.0% and from 0.7% to 1.81%, respectively. Both populations then returned to baseline levels at Ex60. Both exhibited high CX3CR1 expression, as well as expression of CCR5 and CXCR2, with Pop 5 showing high CD28 expression, whereas Pop 6 was CD28 negative. Only one population showed a higher proportion at Ex60 (27.5%) than at Bsl (24.2%): Pop 2, a CD28^+^, CX3CR1-, CCR2-, CCR5- cluster. All clusters were present at comparable proportions and followed similar dynamics in both females and males (Figure S4).

The dynamics of Pop 5 and Pop 6 are consistent with the increased frequency of all CCR5^+^ CD4^+^ T cells of total CD4^+^ T cells at Ex02 (Figure S4). Our data of decreased frequencies of CCR2^+^, CCR5^+^, CX3CR1^+^, and CXCR6^+^ CD4^+^ T cells at Ex60 indicate preferential egress of these cells to peripheral tissues or a downregulation of chemokine receptor expression in those cells retained in circulation.

### 3.4. HIIT-Induced Changes in Chemokine Receptor Expression in γδ T Cells

**Figure 3:**
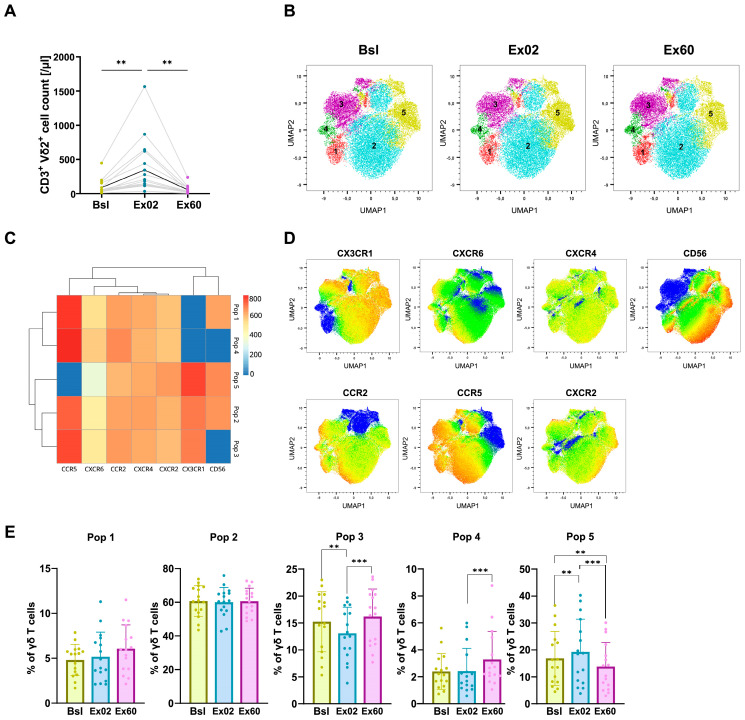
HIIT mobilizes CX3CR1^+^ CD56^+^ γδ T cells. (**A**) γδ cell counts per µL of whole blood before (Bsl), immediately after (Ex02) and one hour after exercise (Ex60), using flow cytometric TBNK kit whole blood staining. (**B**–**E**) UMAP dimensionality reduction and FlowSOM unsupervised clustering were performed using a single integrated dataset comprising CD3^+^ Vδ2^+^ cells from all participants and timepoints based on expression of CD56 and the chemokine receptors CCR5, CXCR2, CXCR6, CX3CR1, CCR2 and CXCR4. (**B**) UMAP visualization of the clusters identified via the FlowSOM clustering algorithm, shown separately at Bsl, Ex02 and Ex60. (**C**) Heatmap showing the surface expression profiles across the five identified clusters. (**D**) UMAP overlaid with marker mean fluorescence intensity (MFI) of cells from all participants and timepoints. (**E**) Population frequency at Bsl, Ex02 and Ex60. Shown are individual values, and bar graphs show means and SD. Percentage data were logit-transformed before being analyzed using repeated-measures ANOVA. Significance levels are indicated by asterisks on the graphs: * *p* ≤ 0.05, ** *p* ≤ 0.01, *** *p* ≤ 0.001, **** *p* ≤ 0.0001. N = 16.

Our panel was designed to detect changes in circulating Vδ2 T cells, since they are the most abundant γδ T cell subset in peripheral blood. As also described by previous studies, Vδ T cells were very responsive to exercise-induced mobilization. Their absolute counts increased 4.2-fold at Ex02 (2.5–9.1, *p* < 0.01), then returned to baseline levels after 1 h of recovery (Figure 3A). Unsupervised clustering based on the chemokine receptors CCR2, CCR5, CXCR2, CXCR4, CXCR6, and CX3CR1 and the cytotoxicity marker CD56 [22] identified five distinct clusters (Figure 3B–D).

At Ex02 Pop 5 exclusively showed an increase from 16.9% out of all γδ T cells to 19.3% and decreased below baseline levels to 13.8% at Ex60. Pop 5 showed the highest CX3CR1 expression out of all clusters and was positive for the cytotoxicity marker CD56. While Vδ2 T cells typically express high levels of CCR5, Pop 5 was negative for CCR5. Pop 3 and Pop 4 were the only clusters increasing at Ex60, which could indicate retention in the periphery. Interestingly, those were the only clusters negative for CD56. No sex-specific differences in cluster proportions or dynamics were detected (Figure S5).

Taken together, the frequency of CXCR4^+^ γδ T cells of total γδ T cells increases with acute HIIT, increasing further at Ex60. CXCR6^+^ γδ T cells and CCR5^+^ γδ T cells increase in frequency at Ex60 compared to Ex02, suggesting that these are not extravasated after acute HIIT (Figure S5).

### 3.5. HIIT-Induced Changes in Chemokine Receptor Expression in Monocytes

**Figure 4:**
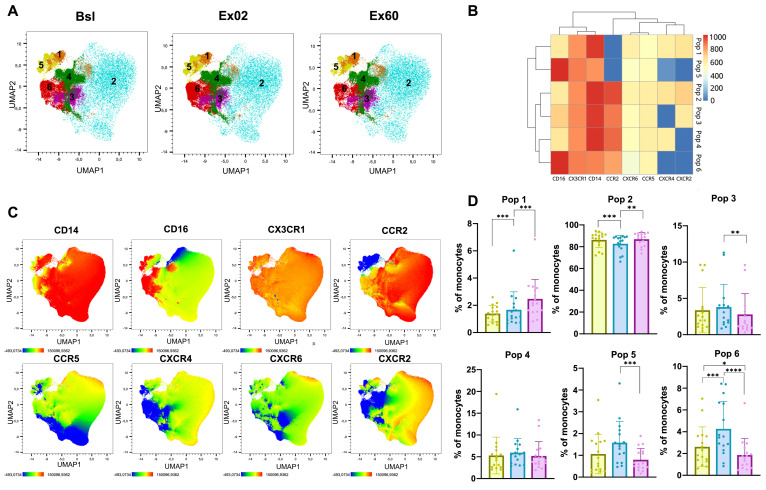
HIIT increases intermediate and non-classical monocyte proportions. UMAP dimensionality reduction and FlowSOM unsupervised clustering were performed using a single integrated dataset comprising CD3^−^ CD14^+^ cells from all participants and timepoints on expression of CD14, CD16, CCR5, CXCR2, CXCR6, CX3CR1, CCR2, and CXCR4. (**A**) UMAP visualization of the clusters identified via the FlowSOM clustering algorithm, shown separately at Bsl (baseline), Ex02 (immediately after exercise) and Ex60 (one hour after exercise). (**B**) Heatmap showing the surface expression profiles across the six identified clusters. (**C**) UMAP overlaid with marker mean fluorescence intensity (MFI) of cells from all participants and timepoints. (**D**) Population frequency at Bsl, Ex02 and Ex60. Shown are individual values, and bar graphs show mean and SD. Percentage data were logit-transformed before being analyzed using repeated-measures ANOVA. Significance levels are indicated by asterisks on the graphs: * *p* ≤ 0.05, ** *p* ≤ 0.01, *** *p* ≤ 0.001, **** *p* ≤ 0.0001. N = 16.

The unsupervised clustering of monocytes based on CD14, CD16, CXCR2, CXCR4, CXCR6, CX3CR1, CCR2, and CCR5 expression revealed six different clusters (Figure 4A–C). The most abundant population, Pop 2, comprised classical monocytes (CD14^+^ CD16^−^) with high CCR2 expression and a moderate expression of the remaining cytokine receptors (Figure 4D). Pop 2 decreased at Ex02 (82.7%) compared to Bsl (86.3%), indicating that other populations could be preferentially mobilized into the circulation. CX3CR1 was highly expressed across all clusters. Pop 6, a population of intermediate monocytes (CD14^+^ CD16^+^), increased at Ex02 (4.3%) compared to Bsl (2.6%) and decreased at Ex60 (1.9%) to lower levels than baseline. Pop 5, consisting of non-classical monocytes (CD14^dim^CD16^+^), also showed the tendency to increase from Bsl (1.1%) to Ex02 (1.6%) and then significantly decreased to 0.8% at Ex60. All clusters exhibited similar abundances and dynamics in females and males (Figure S6).

Overall, we observed that proportions of intermediate and non-classical monocytes increased at Ex02 to then return to baseline or below baseline levels (Figure S6). However, none of the studied chemokine receptors seemed to be specifically expressed in the mobilized populations.

When analyzing single markers, a temporary increase in the percentage of CCR2^+^ and CXCR2^+^ in intermediate monocytes was observed (Figure S7). In non-classical monocytes, CCR5 showed an increase in both frequency of positive cells and MFI at Ex02 and Ex60 (Figure S8).

## 4. Discussion

Understanding the interaction between exercise, immune cell mobilization, and chemokine receptors may pave the way to optimizing exercise-based interventions to boost immune competence and improve health outcomes in situations including, but not limited to, cancer therapy, obesity, infectious diseases, and chronic inflammation. In this study, all participants exhibited strong mobilization of NK cells, γδ T cells, and CD4^+^ T cells in response to one bout of HIIT, consistent with previous studies [7,23,24]. We observed rapid, subset-specific changes in surface chemokine receptor expression, in response to a single bout of HIIT, with cell-type-specific chemokine receptor signatures.

Given the studied time interval of one hour, observed changes in chemokine receptor expression on circulating leukocyte subsets mainly reflect effects of rapid cell redistribution and, to a lesser extent, receptor internalization, rather than transcriptional regulation and de novo protein synthesis [25]. Thus, the changes in chemokine receptor-expressing cell fractions directly inform about predominant cell mobilization and egression.

NK cells were the most responsive immune cell type to exercise-mediated mobilization and exhibited the largest changes in chemokine receptor expression. Our 6.6-fold increase (3.2–18.3) falls within previously reported values, which span from 3-fold [23] to 10-fold [24]. The differences in mobilization levels between different studies highlight the impact of the exercise regimen (endurance [23] vs. incremental [24]) and importance of methodological standards (blood sample timing, processing the blood according to clinically validated protocols) on immune cell quantification [20]. Clustering analysis showed that HIIT preferentially mobilized Pop 1, which was composed mainly of CD56^dim^ NK cells co-expressing CX3CR1^+^ and CXCR2^+^. This observation is consistent with previous studies showing that exercise or acute stress mobilizes preferentially mature cytotoxic (CD56^dim^) NK cells [7,26,27,28]. Recently, Lachota et al. specifically reported increased CX3CR1 and CXCR2 expression in mature NK cells [29], further supporting our observations.

After the initial mobilization phase, circulating NK cell counts declined below baseline, which is believed to reflect deployment to peripheral tissues. This redistribution may enhance NK cell-mediated immune surveillance, as indicated by mouse studies showing that voluntary exercise reduces tumor growth and promotes NK cell infiltration into tumors [30]. Only a limited number of studies have investigated the chemokine receptors mediating NK cell tissue infiltration. Simultaneous CX3CR1 and CXCR2 ligation has been associated with improved NK cell migration [29]. CX3CR1, together with endothelium-bound CX3CL1, has been described to act as an adhesion molecule on cytotoxic effector lymphocytes, thereby governing firm adhesion and trafficking across the endothelium [31].

Another receptor associated with improved tumor infiltration is CCR2, and recent studies have been exploring the potential of CCR2^+^ NK cells. However, in our study, Pop 4, characterized by high CCR2 expression, was not preferentially mobilized by exercise. In contrast, the clusters that increased at Ex60 (Pop 2 and Pop 3) were CD56^bright^, consistent with a cytokine-producing phenotype [32]. CXCR6 expression was very low, in agreement with previous findings that CXCR6^+^ NK cells constitute only a minor fraction of peripheral blood NK cells, specifically within CD56^bright^ subsets, which was also true for our study [33]. In conclusion, HIIT preferentially mobilized CD56^dim^ NK cells displaying a mature, cytotoxic phenotype with CX3CR1 and CXCR2 expression, suggesting increased tissue-infiltrating potential.

HIIT also increased peripheral CD4^+^ and γδ T cell counts immediately after exercise. Previous studies have reported preferential exercise-mediated mobilization of Th1, Th17, and Tregs over Th2 CD4^+^ T cells [34]. In our study, at Ex02, two populations (Pop 5 and Pop 6) increased, suggesting the selective recruitment of these cells. Notably, Pop 6 was CX3CR1^hi^, a phenotype that has been previously associated with effector Th1 [35] and cytotoxic CD4^+^ T cells [36]. Moreover, both Pop 5 and Pop 6 were CCR5^+^. Given the involvement of CCR5 in trafficking to inflamed tissues or tumors, these cells may contribute to enhanced immune surveillance and antitumoral effects [37,38]. Pop 5 was CD28^+^ and CCR7^low^, indicating they may consist of naïve and/or central memory T cells [39]. As our analysis prioritized chemokine receptor expression, memory markers such as CD45RA or CD27 were not included, limiting the definition of memory subsets. Moreover, CXCR6^+^ CD4^+^ T cells have been described as highly cytotoxic [40,41]. Although only a small fraction of these cells was detected, their reduced frequency at Ex60 indicates potential egress into peripheral tissues. This likely corresponds to the decrease in Pop 1 at Ex60, which expressed CXCR6^+^, albeit with low MFI.

γδ T cells are highly sensitive to exercise-induced mobilization. However, to our knowledge, no studies have examined the role of chemokine receptor expression in this process. Chemokine receptors on γδ T cells have been mainly assessed in rheumatoid arthritis and multiple sclerosis, where CXCR3 and CCR5 have been identified as key mediators of γδ T cell migration [42,43]. Our results showed a preferential mobilization of Pop 5 at Ex02, followed by a decrease below baseline levels. Pop 5 showed the highest CX3CR1 expression out of all γδ clusters. Similar to NK cells, CX3CR1 expression has been associated with enhanced endothelial adhesion and tissue infiltration of γδ T cells, via interactions with its ligand CX3CL1 expressed on inflamed vasculature and within tumors [31]. Moreover, Pop 5 was positive for CD56, which is linked to cytotoxic activity of γδ T cells [22]. Collectively, these observations could indicate mobilization of γδ T cells with phenotypes previously associated with extravasation-related pathways and cytotoxicity, warranting further functional investigation. Despite CCR5 being typically expressed at high levels in Vδ2 cells, Pop 5 was CCR5^−^. A previous study described that 80% of Vδ2 was CCR5^+^ in RA patients, which could be lower in healthy donors [43]. CCR5 downregulation has been related to antigen exposure [11]; however, the functional implications of this downregulation remain unclear.

Regarding monocytes, we observed an increase in the fraction of intermediate monocytes at Ex02 and a decrease in both non-classical and intermediate subsets at Ex60. Previous studies have shown that non-classical monocytes drive exercise-induced monocytosis and have linked this process to adrenergic signaling [6,26,44]. Other reports have described increases in intermediate but not classical or non-classical monocytes [45]. Such discrepancies may result from differences in exercise regimens or sampling timepoints. Intermediate monocytes are potent antigen presenters, commonly referred to as inflammatory monocytes, whereas non-classical monocytes are well known for their patrolling and vascular repair functions [46,47,48]. Despite their patrolling function under homeostatic conditions, non-classical monocytes can extravasate and be recruited into inflammation sites [48,49]. Two studies have reported reduced CCR2 on classical monocytes and reduced CCR2 and CXCR2 on intermediate monocytes after moderate endurance exercise [50,51]; however, we observed a transient increase in CCR2^+^ and CXCR2^+^ intermediate monocytes at Ex02 that returned to baseline at Ex60. By contrast, repeated HIIT has been shown to increase CCR5 expression on monocytes [51], which appears consistent with our observation of higher CCR5 expression on non-classical monocytes after a single acute session. CCR5 is involved in monocyte migration [38]; therefore, an exercise-induced increase in its expression in non-classical monocytes could lead to an increased migratory potential of this specific subset.

There are limitations to this study, one being that we focused on a limited set of chemokine receptors; other receptors, such as CXCR3, could also have been relevant. CXCR3 is crucial for lymphocyte vascular transmigration [52] and plays an important role in exercise-induced immune mobilization, specifically of NK cells [53]. Secondly, although we observed distinct chemokine receptor expression patterns, the functional implications of these changes, such as effects on cytotoxicity or infiltration capacity, were not evaluated. Consequently, the link between receptor expression and effector function remains indirect. Generalizability might be limited because of the small sample size, unequal numbers of male vs. female participants, and a certain age range; however, stratified exploratory analyses indicated comparable cluster abundances and temporal trends in female and male participants. Dietary intake was standardized in the two hours preceding exercise; however, dietary factors beyond this period may represent a source of variability in immune parameters. Finally, this study examined a single bout of HIIT, thereby capturing acute changes in the immune compartment. Future research should explore the effects of different exercise protocols with varying FITT (frequency, intensity, time, and type) criteria.

## 5. Conclusions

In conclusion, our findings indicate that one bout of HIIT influences immune cell trafficking through multiple chemokine receptor pathways across diverse immune cell subsets. Preferentially mobilized or egressed cells showed expression of chemokine receptors associated with improved endothelial interaction, migratory capacities and cytotoxic potential. Specifically, HIIT preferentially mobilized CD56^dim^ CX3CR1^+^CXCR2^+^ NK cells, CX3CR1^+^ CCR5^+^ CD4^+^ T cells, and CX3CR1^+^ CD56^+^ γδ T cells and increased intermediate and non-classical monocyte proportions. These findings represent phenotypic observations, and further studies are needed to determine functional implications and the specific tissues into which these mobilized leukocytes migrate.

## Figures and Tables

**Table 1:** Participant characteristics in the INHALE study. BMI, body mass index; HR, heart rate; BPM, beats per minute; HIIT, high-intensity interval training; CPET, cardiopulmonary exercise test.

**Participant characteristics**		n = 16
**Age—years**		
	Mean	31.7
	Range	25–65
**Sex—no.**		
	Male	11
	Female	5
**BMI—kg/m^2^**		
	Mean	23
	Range	20.3–26.8
**Fat percentage—%**		
	Mean	22.2
	Range	12.7–31.4
**Smoking status—no.**		
	Never smoker	7
	Former smoker (stopped >= 4 weeks)	4
	Active daily smoker	0
	Active “only on special occasions” smoker	5
**Aerobic capacity (VO_2_peak)—mL/min/kg**		
	Mean	39.7
	Range	29.0–47.3
**Max. Power output—W**		
	Mean	338
	Range	199–490
**Max. HR—bpm**		
	Mean	178
	Range	151–201
**Power output during HIIT relative to CPET—%**		
	Mean	118
	Range	93–137
**HR during HIIT relative to CPET—%**		
	Mean	96
	Range	87–106

## Data Availability

The datasets generated during and/or analyzed during the current study are available from the corresponding author upon reasonable request.

## Supplementary Materials

The following supporting information can be downloaded at https://www.mdpi.com/article/10.3390/biom16020263/s1. Figure S1: INHALE study design; Figure S2: Gating strategies; Figure S3: Population frequency in females and males. Frequency of chemokine receptor-positive NK cells; Figure S4: Population frequency in females and males. Frequency of chemokine receptor-positive CD4^+^ T cells; Figure S5: Population frequency in females and males. Frequency of chemokine receptor-positive γδ cells; Figure S6: Population frequency in females and males. Frequency of chemokine receptor positive cMo; Figure S7: Frequency of chemokine receptor positive intMo; Figure S8: Frequency of chemokine receptor positive ncMo; Table S1: Antibody list. Supplementary Data S1. MFI values underlying the FlowSOM heatmaps.

## Abbreviations

The following abbreviations are used in this manuscript:

BMI	Body mass index
Bsl	Blood samples taken at baseline
CCR	C-C chemokine receptor
CPET	Cardiopulmonary exercise test
CXCR	C-X-C chemokine receptor
CX3CR	C-X3-C chemokine receptor
Ex02	Blood samples taken immediately after exercise
Ex60	Blood samples taken 60 min after exercise
FITT	Frequency, intensity, time and type
HIIT	High-intensity interval training
HR	Heart rate
MFI	Mean fluorescence intensity
NK cells	Natural killer cells
PBMCs	Peripheral blood mononuclear cells
RER	Respiratory exchange ratio
SD	Standard deviation
UMAP	Uniform manifold approximation and projection
VO_2_	Peak oxygen uptake

## References

[1] Ruegsegger G.N., Booth F.W. (2018). Health Benefits of Exercise. Cold Spring Harb. Perspect. Med..

[2] Olofsson G.H., Jensen A.W.P., Idorn M., Straten P.T. (2020). Exercise Oncology and Immuno-Oncology; A (Future) Dynamic Duo. Int. J. Mol. Sci..

[3] Rosa-Neto J.C., Lira F.S., Little J.P., Landells G., Islam H., Chazaud B., Pyne D.B., Teixeira A.M., Batatinha H., Moura Antunes B. (2022). Immunometabolism-Fit: How Exercise and Training Can Modify T Cell and Macrophage Metabolism in Health and Disease. Exerc. Immunol. Rev..

[4] Edwards H.T., Wood W.B. (1932). A Study of Leukocytosis in Exercise. Arbeitsphysiologie.

[5] Krüger K., Lechtermann A., Fobker M., Völker K., Mooren F.C. (2008). Exercise-Induced Redistribution of T Lymphocytes Is Regulated by Adrenergic Mechanisms. Brain Behav. Immun..

[6] Graff R.M., Kunz H.E., Agha N.H., Baker F.L., Laughlin M., Bigley A.B., Markofski M.M., LaVoy E.C., Katsanis E., Bond R.A. (2018). B2-Adrenergic Receptor Signaling Mediates the Preferential Mobilization of Differentiated Subsets of CD8+ T-Cells, NK-Cells and Non-Classical Monocytes in Response to Acute Exercise in Humans. Brain Behav. Immun..

[7] Campbell J.P., Riddell N.E., Burns V.E., Turner M., van Zanten J.J.C.S.V., Drayson M.T., Bosch J.A. (2009). Acute Exercise Mobilises CD8+ T Lymphocytes Exhibiting an Effector-Memory Phenotype. Brain Behav. Immun..

[8] Hughes C.E., Nibbs R.J.B. (2018). A Guide to Chemokines and Their Receptors. FEBS J..

[9] Kohli K., Pillarisetty V.G., Kim T.S. (2022). Key Chemokines Direct Migration of Immune Cells in Solid Tumors. Cancer Gene Ther..

[10] Chen K., Bao Z., Tang P., Gong W., Yoshimura T., Wang J.M. (2018). Chemokines in Homeostasis and Diseases. Cell. Mol. Immunol..

[11] Glatzel A., Wesch D., Schiemann F., Brandt E., Janssen O., Kabelitz D. (2002). Patterns of Chemokine Receptor Expression on Peripheral Blood Γδ T Lymphocytes: Strong Expression of CCR5 Is a Selective Feature of Vδ2/Vγ9 Γδ T Cells1. J. Immunol..

[12] Di Pilato M., Kfuri-Rubens R., Pruessmann J.N., Ozga A.J., Messemaker M., Cadilha B.L., Sivakumar R., Cianciaruso C., Warner R.D., Marangoni F. (2021). CXCR6 Positions Cytotoxic T Cells to Receive Critical Survival Signals in the Tumor Microenvironment. Cell.

[13] Gerlach C., Moseman E.A., Loughhead S.M., Alvarez D., Zwijnenburg A.J., Waanders L., Garg R., de la Torre J.C., von Andrian U.H. (2016). The Chemokine Receptor CX3CR1 Defines Three Antigen-Experienced CD8 T Cell Subsets with Distinct Roles in Immune Surveillance and Homeostasis. Immunity.

[14] Krinninger P., Ensenauer R., Ehlers K., Rauh K., Stoll J., Krauss-Etschmann S., Hauner H., Laumen H. (2014). Peripheral Monocytes of Obese Women Display Increased Chemokine Receptor Expression and Migration Capacity. J. Clin. Endocrinol. Metab..

[15] Barry J.C., Simtchouk S., Durrer C., Jung M.E., Little J.P. (2017). Short-Term Exercise Training Alters Leukocyte Chemokine Receptors in Obese Adults. Med. Sci. Sports Exerc..

[16] Chammas R., Buzaglo G.B.B., Telles G.D., Araújo R.B., Junior G.D.S., Ruberti O.M., Ferreira M.L.V., Derchain S.F.M., Vechin F.C., Conceição M.S. (2024). The Therapeutic Potential of Physical Exercise in Cancer: The Role of Chemokines. Int. J. Mol. Sci..

[17] Idorn M., Thor Straten P. (2018). Chemokine Receptors and Exercise to Tackle the Inadequacy of T Cell Homing to the Tumor Site. Cells.

[18] Leuchte K., Luu T.V., Saló S.F., Madsen K., Heide-Ottosen L., Skadborg S.K., Kemming J.S., Holmström M.O., Chen H., Olsen L.R. (2026). Moving for Optimal Immunity: The Effect of Acute High-Intensity Interval Training on Phenotype, Virus Specificity and Chemokine Receptor Expression in Human CD8+ T Cells. Front. Immunolgy.

[19] Myers J., Arena R., Franklin B., Pina I., Kraus W.E., McInnis K., Balady G.J. (2009). Recommendations for Clinical Exercise Laboratories: A Scientific Statement from the American Heart Association. Circulation.

[20] Olofsson G.H., Mikkelsen M.K., Ragle A.M., Christiansen A.B., Olsen A.P., Ottosen L.H., Horsted C.B., Moon C., Pedersen S., Noerregaard L.E. (2022). High Intensity Aerobic Exercise Training and Immune Cell Mobilization in Patients with Lung Cancer (HI AIM)—A Randomized Controlled Trial. BMC Cancer.

[21] Cooper M.A., Fehniger T.A., Caligiuri M.A. (2001). The Biology of Human Natural Killer-Cell Subsets. Trends Immunol..

[22] Alexander A.A.Z., Maniar A., Cummings J.-S., Hebbeler A.M., Schulze D.H., Gastman B.R., Pauza C.D., Strome S.E., Chapoval A.I. (2008). Isopentenyl Pyrophosphate-Activated CD56+ {gamma}{delta} T Lymphocytes Display Potent Antitumor Activity toward Human Squamous Cell Carcinoma. Clin. Cancer Res..

[23] Gabriel H., Schwarz L., Born P., Kindermann W. (1992). Differential Mobilization of Leucocyte and Lymphocyte Subpopulations into the Circulation during Endurance Exercise. Eur. J. Appl. Physiol. Occup. Physiol..

[24] Anane L.H., Edwards K.M., Burns V.E., Drayson M.T., Riddell N.E., van Zanten J.J.C.S.V., Wallace G.R., Mills P.J., Bosch J.A. (2009). Mobilization of Γδ T Lymphocytes in Response to Psychological Stress, Exercise, and β-Agonist Infusion. Brain Behav. Immun..

[25] Marchese A. (2014). Endocytic Trafficking of Chemokine Receptors. Curr. Opin. Cell Biol..

[26] Dimitrov S., Lange T., Born J. (2010). Selective Mobilization of Cytotoxic Leukocytes by Epinephrine. J. Immunol..

[27] Bosch J.A., Berntson G.G., Cacioppo J.T., Marucha P.T. (2005). Differential Mobilization of Functionally Distinct Natural Killer Subsets during Acute Psychologic Stress. Psychosom. Med..

[28] Timmons B.W., Cieslak T. (2008). Human Natural Killer Cell Subsets and Acute Exercise: A Brief Review. Exerc. Immunol. Rev..

[29] Lachota M., Zielniok K., Palacios D., Kanaya M., Peena L., Hoel H.J., Wiiger M.T., Kveberg L., Hautz W., Zagożdżon R. (2023). Mapping the Chemotactic Landscape in NK Cells Reveals Subset-Specific Synergistic Migratory Responses to Dual Chemokine Receptor Ligation. EBioMedicine.

[30] Pedersen L., Idorn M., Olofsson G.H., Lauenborg B., Nookaew I., Hansen R.H., Johannesen H.H., Becker J.C., Pedersen K.S., Dethlefsen C. (2016). Voluntary Running Suppresses Tumor Growth through Epinephrine- and IL-6-Dependent NK Cell Mobilization and Redistribution. Cell Metab..

[31] Nishimura M., Umehara H., Nakayama T., Yoneda O., Hieshima K., Kakizaki M., Dohmae N., Yoshie O., Imai T. (2002). Dual Functions of Fractalkine/CX3C Ligand 1 in Trafficking of Perforin+/Granzyme B+ Cytotoxic Effector Lymphocytes That Are Defined by CX3CR1 Expression. J. Immunol..

[32] Poli A., Michel T., Thérésine M., Andrès E., Hentges F., Zimmer J. (2009). CD56bright Natural Killer (NK) Cells: An Important NK Cell Subset. Immunology.

[33] Hess L.U., Martrus G., Ziegler A.E., Langeneckert A.E., Salzberger W., Goebels H., Sagebiel A.F., Hagen S.H., Poch T., Ravichandran G. (2020). The Transcription Factor Promyelocytic Leukemia Zinc Finger Protein Is Associated with Expression of Liver-Homing Receptors on Human Blood CD56bright Natural Killer Cells. Hepatol. Commun..

[34] Goldsmith C.D., Donovan T., Vlahovich N., Pyne D.B. (2021). Unlocking the Role of Exercise on CD4+ T Cell Plasticity. Front. Immunol..

[35] Batista N.V., Chang Y.-H., Chu K.-L., Wang K.C., Girard M., Watts T.H. (2020). T Cell-Intrinsic CX3CR1 Marks the Most Differentiated Effector CD4(+) T Cells, but Is Largely Dispensable for CD4(+) T Cell Responses during Chronic Viral Infection. Immunohorizons.

[36] Weiskopf D., Bangs D.J., Sidney J., Kolla R.V., De Silva A.D., de Silva A.M., Crotty S., Peters B., Sette A. (2015). Dengue Virus Infection Elicits Highly Polarized CX3CR1^+^ Cytotoxic CD4^+^ T Cells Associated with Protective Immunity. Proc. Natl. Acad. Sci. USA.

[37] Qin S., Rottman J.B., Myers P., Kassam N., Weinblatt M., Loetscher M., Koch A.E., Moser B., Mackay C.R. (1998). The Chemokine Receptors CXCR3 and CCR5 Mark Subsets of T Cells Associated with Certain Inflammatory Reactions. J. Clin. Investig..

[38] Korbecki J., Grochans S., Gutowska I., Barczak K., Baranowska-Bosiacka I. (2020). CC Chemokines in a Tumor: A Review of Pro-Cancer and Anti-Cancer Properties of Receptors CCR5, CCR6, CCR7, CCR8, CCR9, and CCR10 Ligands. Int. J. Mol. Sci..

[39] Okada R., Kondo T., Matsuki F., Takata H., Takiguchi M. (2008). Phenotypic Classification of Human CD4+ T Cell Subsets and Their Differentiation. Int. Immunol..

[40] Shi S., Xing H., Xu X., Chai J., Lu Z., Wang J., Wang B. (2024). CXCR6 Defines Therapeutic Subtypes of CD4+ Cytotoxic T Cell Lineage for Adoptive Cell Transfer Therapy in Pediatric B Cell Acute Lymphoblastic Leukemia. Int. Immunopharmacol..

[41] Hou L., Yuki K. (2022). CCR6 and CXCR6 Identify the Th17 Cells with Cytotoxicity in Experimental Autoimmune Encephalomyelitis. Front. Immunol..

[42] Murzenok P.P., Matusevicius D., Freedman M.S. (2002). Gamma/Delta T Cells in Multiple Sclerosis: Chemokine and Chemokine Receptor Expression. Clin. Immunol..

[43] Mo W.X., Yin S.S., Chen H., Zhou C., Zhou J.X., Zhao L.D., Fei Y.Y., Yang H.X., Guo J.B., Mao Y.J. (2017). Chemotaxis of Vδ 2 T Cells to the Joints Contributes to the Pathogenesis of Rheumatoid Arthritis. Ann. Rheum. Dis..

[44] Koivula T., Lempiäinen S., Neuvonen J., Norha J., Hollmén M., Sundberg C.J., Rundqvist H., Minn H., Rinne P., Heinonen I. (2024). The Effect of Exercise and Disease Status on Mobilization of Anti-Tumorigenic and pro-Tumorigenic Immune Cells in Women with Breast Cancer. Front. Immunol..

[45] Koivula T., Lempiäinen S., Rinne P., Rannikko J.H., Hollmén M., Sundberg C.J., Rundqvist H., Minn H., Heinonen I. (2023). The Effect of Acute Exercise on Circulating Immune Cells in Newly Diagnosed Breast Cancer Patients. Sci. Rep..

[46] Mukherjee R., Kanti Barman P., Kumar Thatoi P., Tripathy R., Kumar Das B., Ravindran B. (2015). Non-Classical Monocytes Display Inflammatory Features: Validation in Sepsis and Systemic Lupus Erythematous. Sci. Rep..

[47] Tahir S., Steffens S. (2021). Nonclassical Monocytes in Cardiovascular Physiology and Disease. Am. J. Physiol. Cell Physiol..

[48] Guglietta S., Krieg C. (2023). Phenotypic and Functional Heterogeneity of Monocytes in Health and Cancer in the Era of High Dimensional Technologies. Blood Rev..

[49] Auffray C., Fogg D., Garfa M., Elain G., Join-Lambert O., Kayal S., Sarnacki S., Cumano A., Lauvau G., Geissmann F. (2007). Monitoring of Blood Vessels and Tissues by a Population of Monocytes with Patrolling Behavior. Science.

[50] Blanks A.M., Pedersen L.N., Bohmke N., Mihalick V.L., Franco R.L. (2022). Sex Differences in Monocyte CCR2 Expression and Macrophage Polarization Following Acute Exercise. Life Sci..

[51] Hong S., Mills P.J. (2008). Effects of an Exercise Challenge on Mobilization and Surface Marker Expression of Monocyte Subsets in Individuals with Normal vs. Elevated Blood Pressure. Brain Behav. Immun..

[52] Mikucki M.E., Fisher D.T., Matsuzaki J., Skitzki J.J., Gaulin N.B., Muhitch J.B., Ku A.W., Frelinger J.G., Odunsi K., Gajewski T.F. (2015). Non-Redundant Requirement for CXCR3 Signalling during Tumoricidal T-Cell Trafficking across Tumour Vascular Checkpoints. Nat. Commun..

[53] Parent-Roberge H., Fontvieille A., Poirier L., Tai L.-H., Pavic M., Fülöp T., Riesco E. (2024). Acute Natural Killer Cells Response to a Continuous Moderate Intensity and a Work-Matched High Intensity Interval Exercise Session in Metastatic Cancer Patients Treated with Chemotherapy. Brain Behav. Immun. Health.

