# Supplementary material for: Chemokine Receptor Profile of Circulating Leukocyte Subsets in Response to Acute High-Intensity Interval Training
Source: Biomolecules. 2026 Feb 7;16(2):263. doi: 10.3390/biom16020263 (PMC12937720; doi:10.3390/biom16020263)
Supplement: Supplementary file 1 [file biomolecules-16-00263-s001.zip › Supplementary Figures.pdf]

Fig. S1

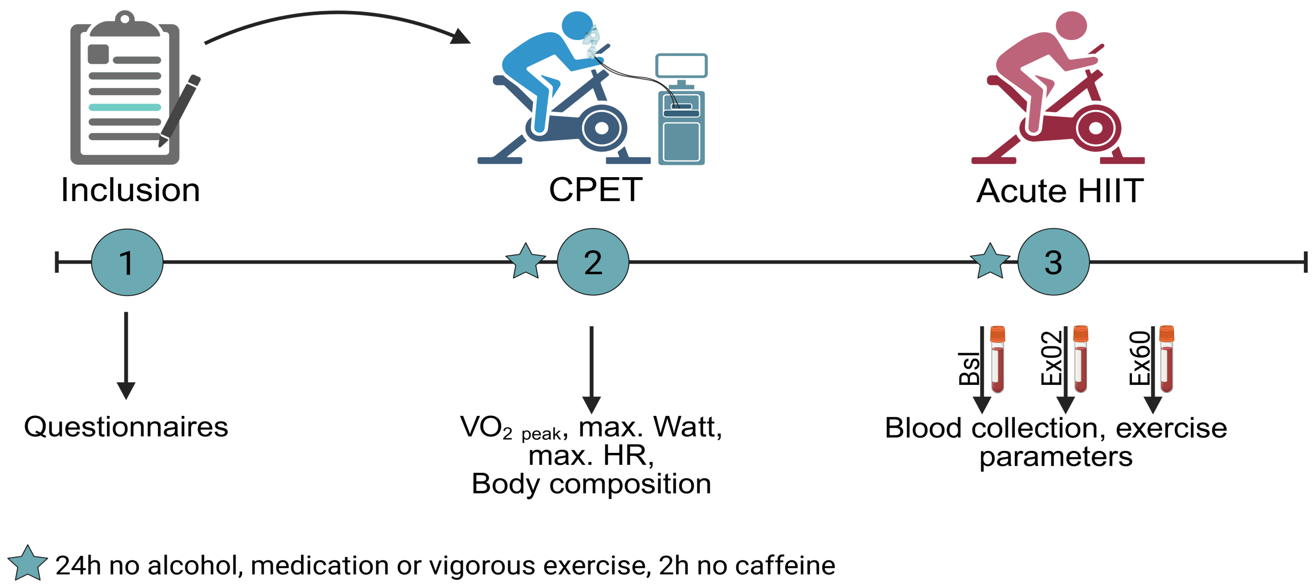

S1. INHALE study design. CPET, cardiopulmonary exercise test; HIIT, high-intensity interval training; HR, heart rate. Created with Biorender.

Fig. S2

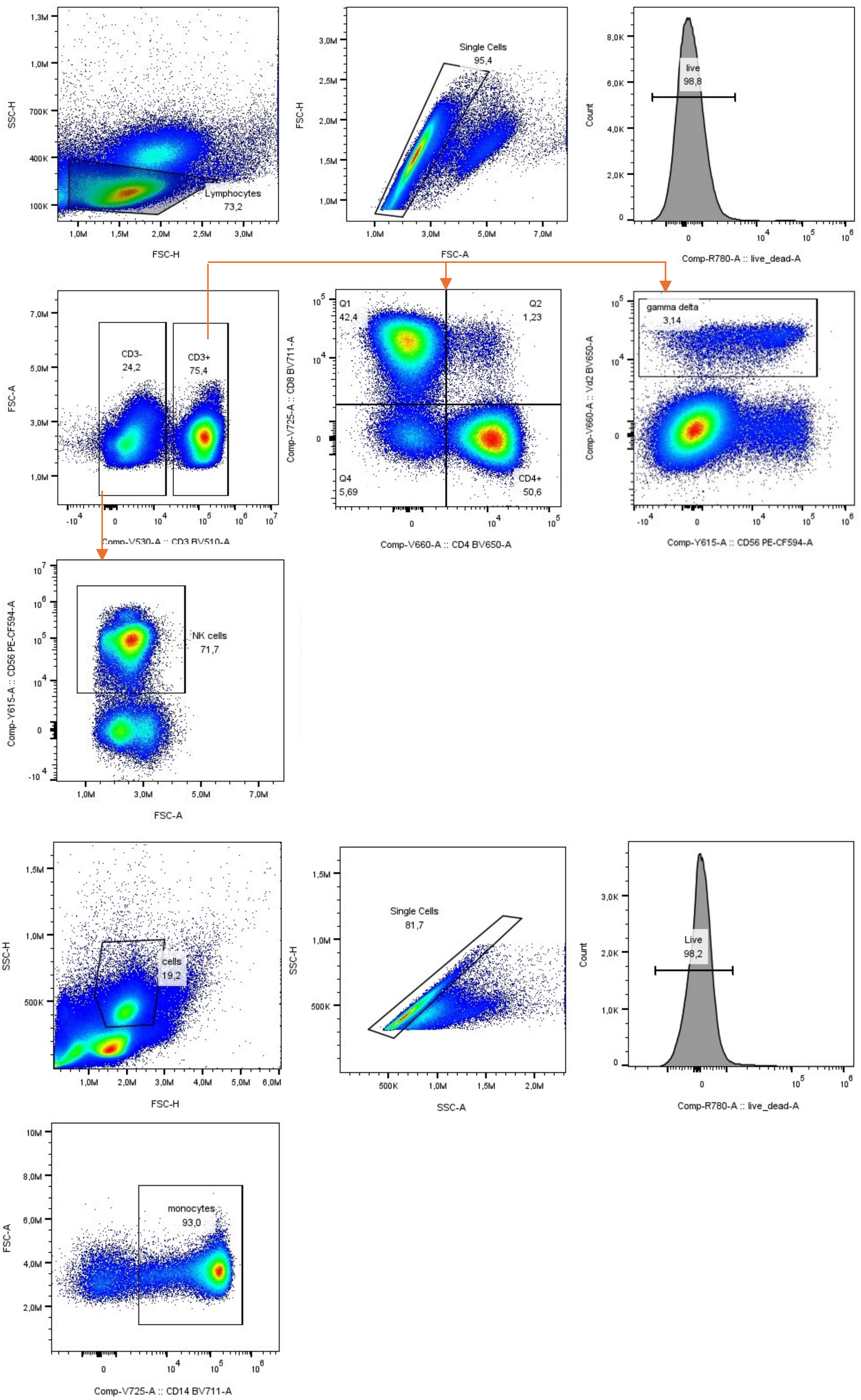

Fig S2. Flow cytometry gating strategies for NK, γδ, CD4+ T cells and monocytes.

Fig. S3

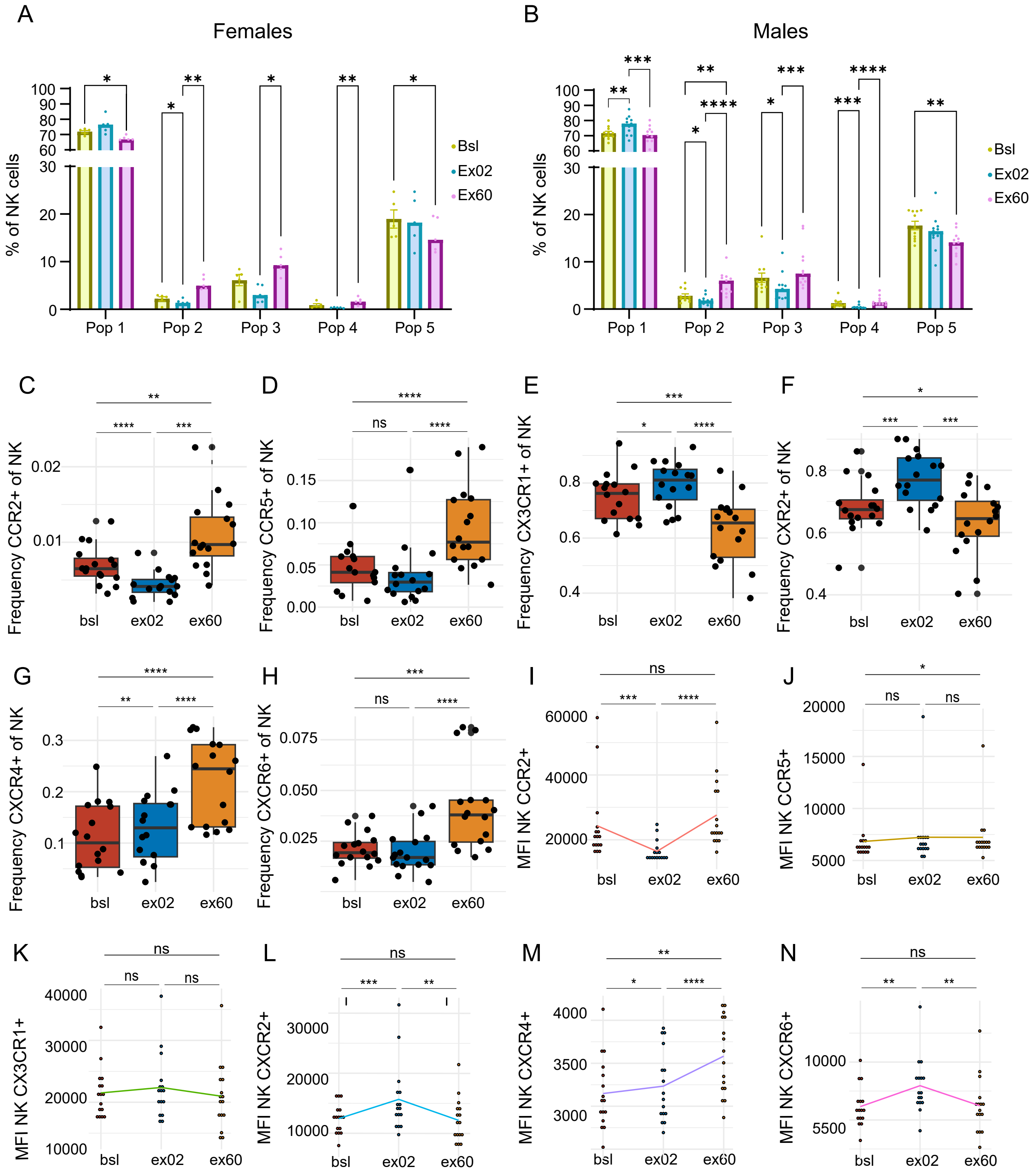

S3. (A-B) Population frequency at baseline (Bsl), immediately after (Ex02) and one hour after exercise (Ex60) separated by sex. Percentage data were logit-transformed before being analyzed using repeated-measures ANOVA. (C-H) Frequency of NK cells positive for CCR2, CCR5, CX3CR1, CXCR2, CXCR4, and CXCR6 at Bsl, Ex02, and Ex60. (I-N) Median fluorescence intensity (MFI) of chemokine receptors. Statistical analysis was performed using paired Wilcoxon tests with Holm-adjustment for multiple testing. Significance levels indicated by asterisks on the graphs: ns $p > 0.05$, * $p \leq 0.05$, ** $p \leq 0.01$, *** $p \leq 0.001$, **** $p \leq 0.0001$. N= 16.

Fig. S4

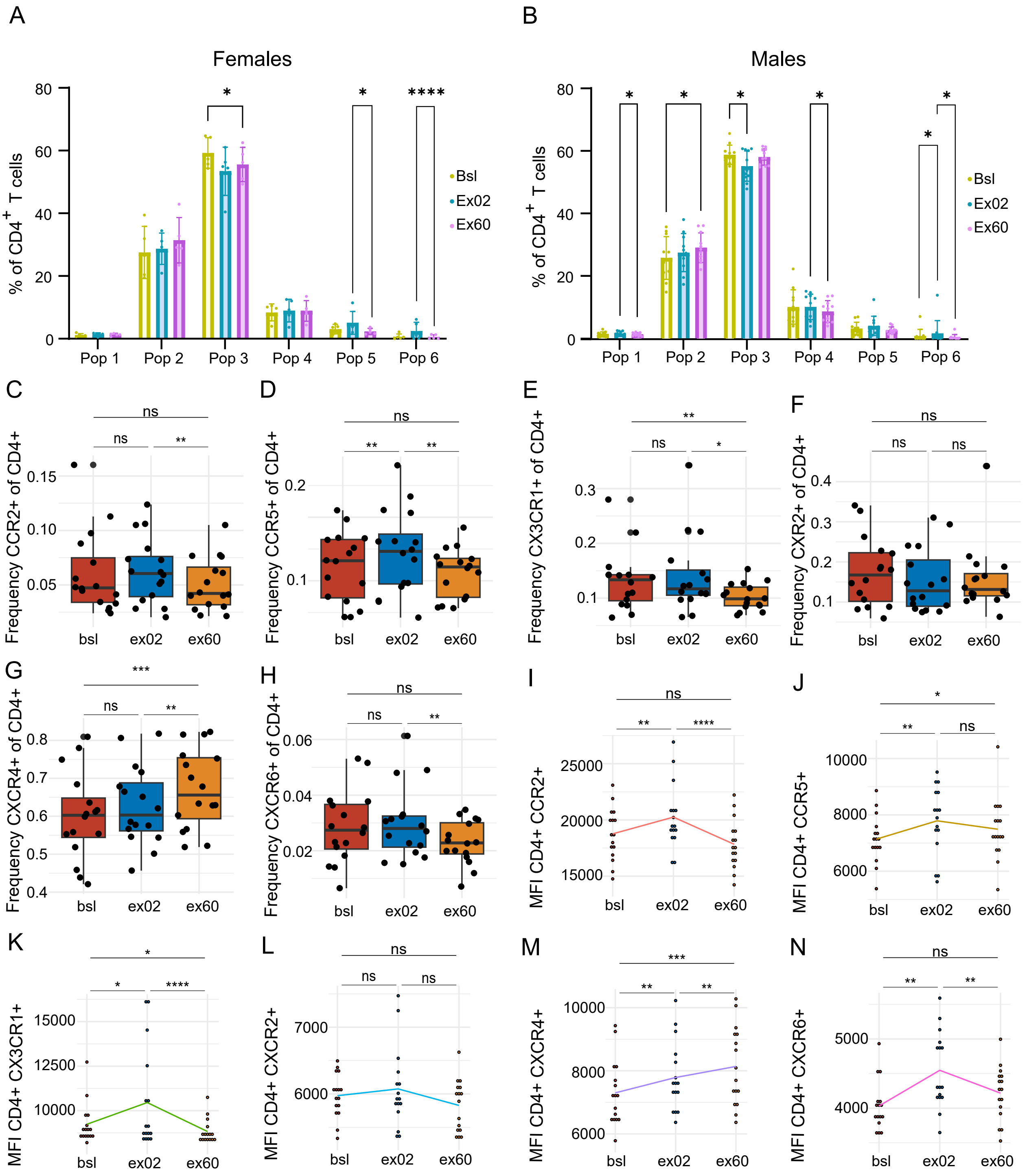

S4. (A-B) Population frequency at baseline (Bsl), immediately after (Ex02) and one hour after exercise (Ex60) separated by sex. Percentage data were logit-transformed before being analyzed using repeated-measures ANOVA. (C-H) Frequency of CD4+ T cells positive for CCR2, CCR5, CX3CR1, CXCR2, CXCR4, and CXCR6 at Bsl, Ex02, and Ex60. (I-N) Median fluorescence intensity (MFI) of chemokine receptors. Statistical analysis was performed using paired Wilcoxon tests with Holm-adjustment for multiple testing. Significance levels indicated by asterisks on the graphs: ns p> 0.05, * p ≤ 0.05, ** p ≤ 0.01, *** p ≤ 0.001, **** p ≤ 0.0001. N= 16.

Fig. S5

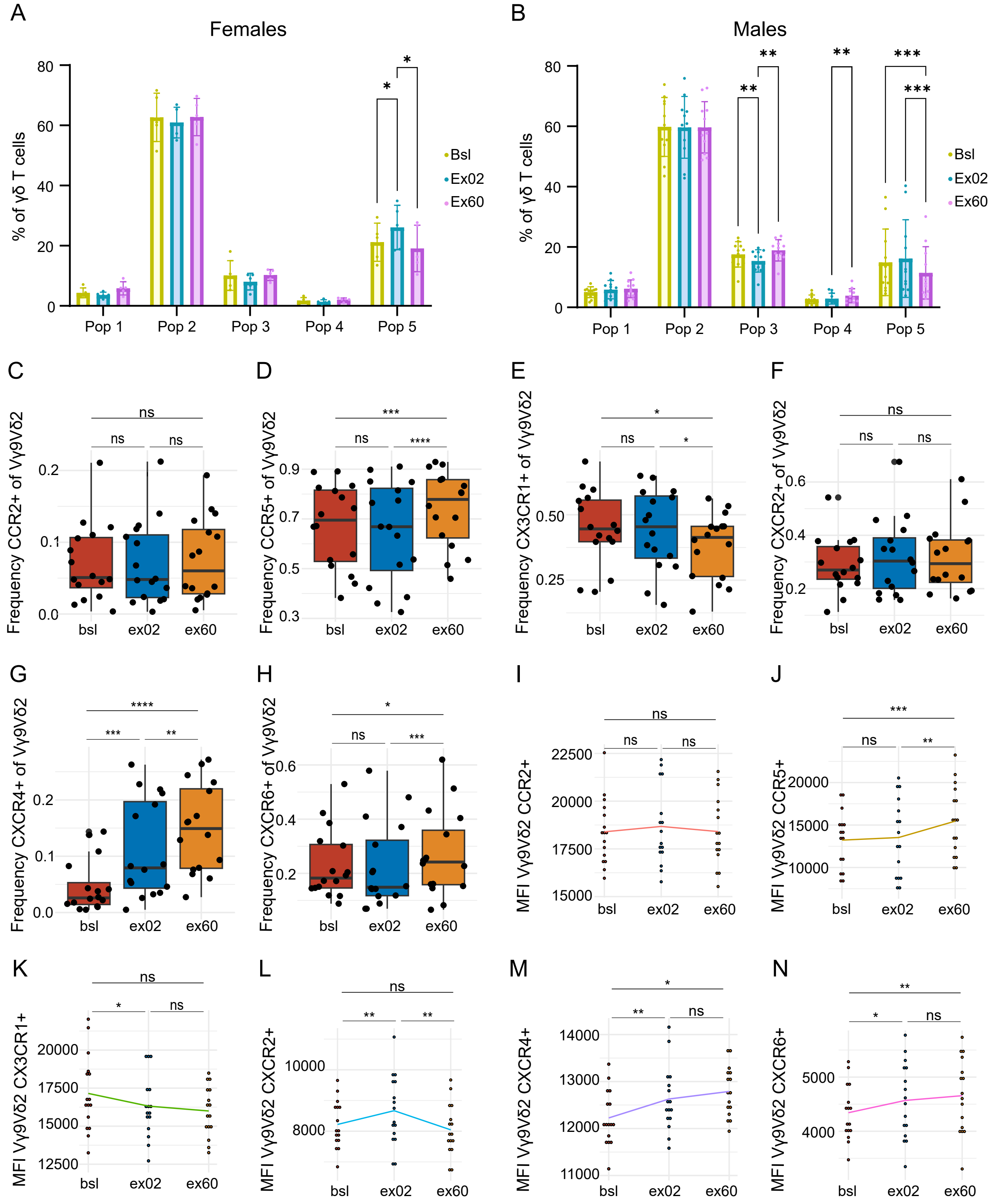

S5. (A-B) Population frequency at baseline (Bsl), immediately after (Ex02) and one hour after exercise (Ex60) separated by sex. Percentage data were logit-transformed before being analyzed using repeated-measures ANOVA. (C-H) Frequency of γδ cells positive for CCR2, CCR5, CX3CR1, CXCR2, CXCR4, and CXCR6 at Bsl, Ex02, and Ex60. (I-N) Median fluorescence intensity (MFI) of chemokine receptors. Statistical analysis was performed using paired Wilcoxon tests with Holm-adjustment for multiple testing. Significance levels indicated by asterisks on the graphs: ns $p > 0.05$, * $p \leq 0.05$, ** $p \leq 0.01$, *** $p \leq 0.001$, **** $p \leq 0.0001$. N= 16.

Fig. S6

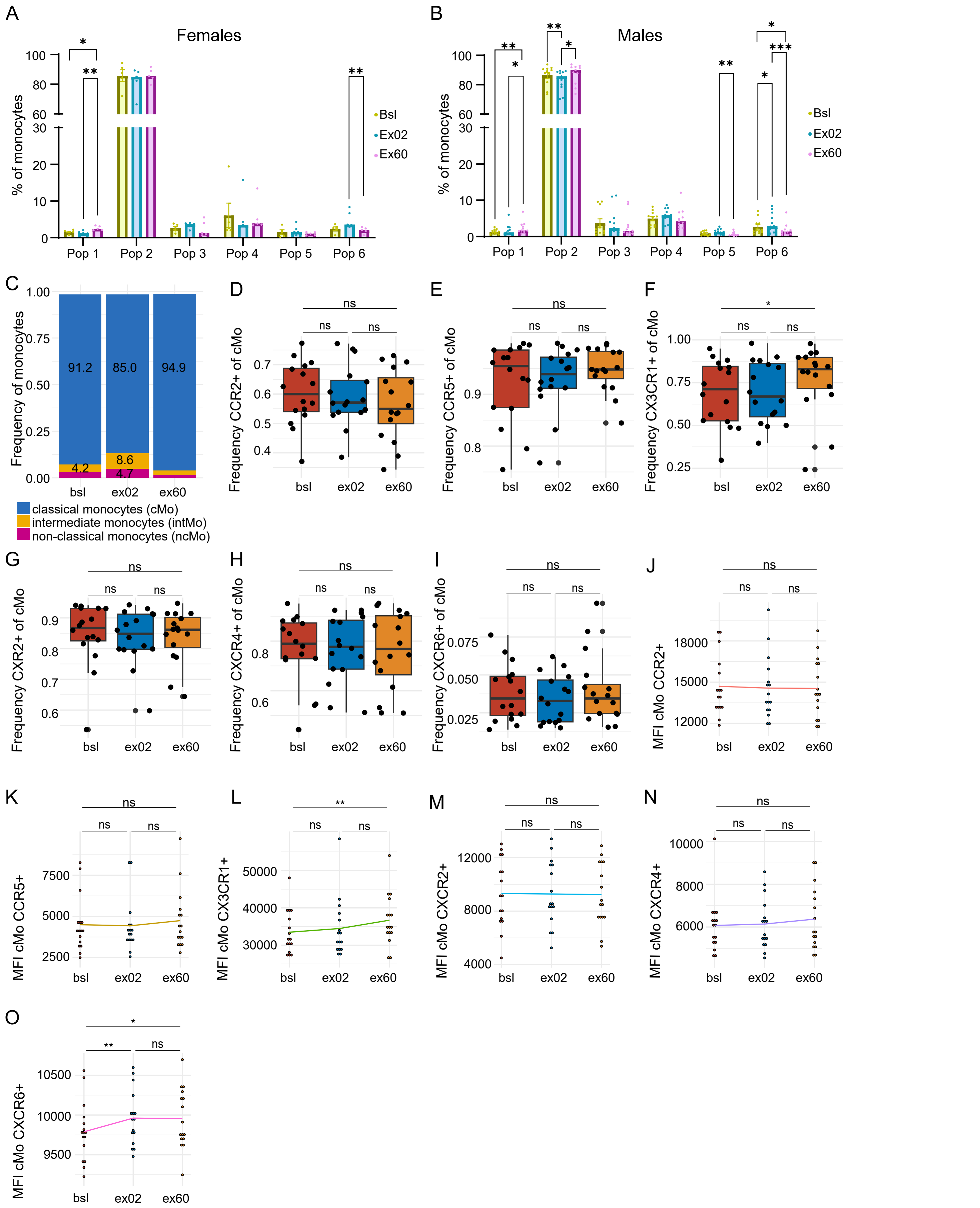

S6. (A-B) Population frequency at baseline (Bsl), immediately after (Ex02) and one hour after exercise (Ex60) separated by sex. Percentage data were logit-transformed before being analyzed using repeated-measures ANOVA. C) Proportions of Classical (cMo; CD14+ CD16-), intermediate (intMo; CD14+CD16+) and non-classical monocytes (ncMo; CD14+ CD16++). (D-I) Frequency of cMo positive for CCR2, CCR5, CX3CR1, CXCR2, CXCR4, and CXCR6 at Bsl, Ex02, and Ex60. (J-O) Median fluorescence intensity (MFI) of chemokine receptors. Statistical analysis was performed using paired Wilcoxon tests with Holm-adjustment for multiple testing. Significance levels indicated by asterisks on the graphs: ns $p > 0.05$, * $p \leq 0.05$, ** $p \leq 0.01$, *** $p \leq 0.001$, **** $p \leq 0.0001$. N= 16.

Fig. S7

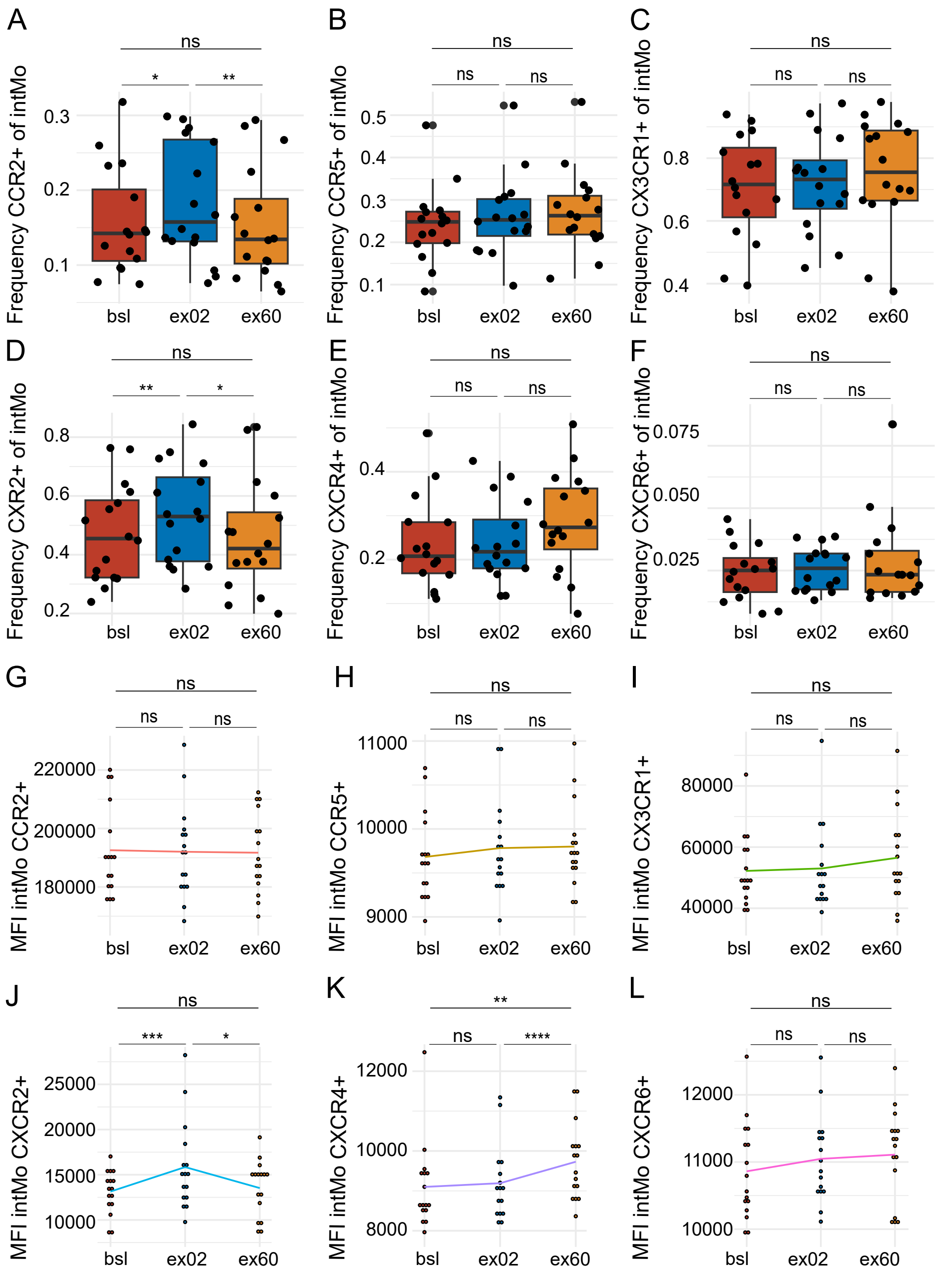

S7. (A-F) Frequency of intMo positive for CCR2, CCR5, CX3CR1, CXCR2, CXCR4, and CXCR6 at baseline (bsl), immediately after (ex02), and one hour after exercise (ex60). (G-L) Median fluorescence intensity (MFI) of chemokine receptors. Statistical analysis was performed using paired Wilcoxon tests with Holm-adjustment for multiple testing. Significance levels indicated by asterisks on the graphs: ns p> 0.05, * p ≤ 0.01, *** p ≤ 0.001, **** p ≤ 0.0001. N= 16.

Fig. S8

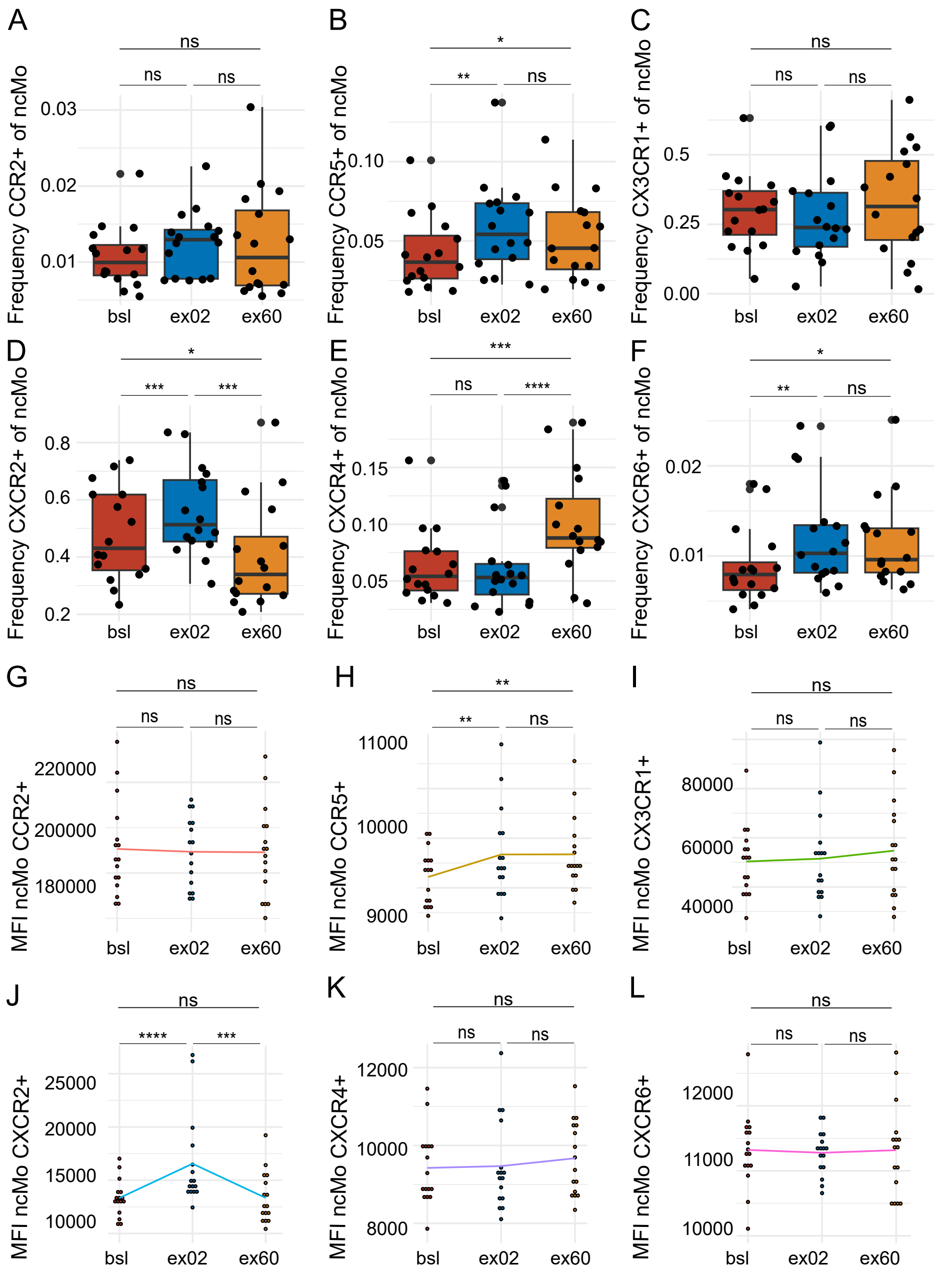

S8. (A-F) Frequency of ncMo positive for CCR2, CCR5, CX3CR1, CXCR2, CXCR4, and CXCR6 at baseline (bsl), immediately after (ex02), and one hour after exercise (ex60). (G-L) Median fluorescence intensity (MFI) of chemokine receptors. Statistical analysis was performed using paired Wilcoxon tests with Holm-adjustment for multiple testing. Significance levels indicated by asterisks on the graphs: ns p> 0.05, * p ≤ 0.01, *** p ≤ 0.001, **** p ≤ 0.0001. N= 16.
