# Supplementary material for: Chemokine Receptor Profile of Circulating Leukocyte Subsets in Response to Acute High-Intensity Interval Training
Source: Biomolecules. 2026 Feb 7;16(2):263. doi: 10.3390/biom16020263 (PMC12937720; doi:10.3390/biom16020263)
Supplement: Supplementary file 1 [file biomolecules-16-00263-s001.zip › supplementary tables.pdf]

Table S1. Antibody list

NAME	SUPPLIER	REAC-TIVITY	CLONE	CAT. NUMBER	VOLUME (/50 µl)
**CD192 (CCR2)-BV421**	BD Biosci-ences	Human	48607	564067	1.00
**CD56-PECF594**	BD Biosci-ences	Human	NCAM16.2	564849	0.50
**CD3ε-BV510**	BD Biosci-ences	Human	UCHT1	563109	1.00
**CCR7 (CD197)-RB780**	BD Biosci-ences	Human	2-L1-A	568749	2.50
**CD195 (CCR5)-BV605**	BD Biosci-ences	Human	2D7/CCR5	563379	2.50
**CD16 (FcγRIII)-BV650**	BD Biosci-ences	Human	3G8	563691	1.00
**CD28 (TLR2)-PECF594**	BD Biosci-ences	Human	CD28.2	562323	0.25
**CD181 (CXCR2)-RY586**	BD Biosci-ences	Human	6C6	753253	2.50
**CD184 (CXCR4)-APC**	BD Biosci-ences	Human	12G5	560936	0.50
**CD14-BV711-**	BD Biosci-ences	Human	RPA-T8	563373	1.00
**CD4-BV650**	BD Biosci-ences	Human	12G5	563876	0.10
**CD186 (CXCR6)-BB700**	BD Biosci-ences	Human	MPHIP9 (also known as MφP-9)	745882	2.50
**CX3CR1-BB515**	BD Biosci-ences	Human	SK3 (also known as Leu3a)	565902	2.50
**Vδ2-BV650**	BD Biosci-ences	Human	B6	743752	0.50
**LIVE/DEAD™ Fixable Near-IR Dead Cell Stain**	Fisher Scien-tific	-	-	L23105	0.01
